# Bio-Insecticidal Potential of Nucleopolyhedrovirus and Granulovirus Mixtures to Control the Fall Armyworm *Spodoptera frugiperda* (J.E. Smith, 1797) (Lepidoptera: Noctuidae)

**DOI:** 10.3390/v11080684

**Published:** 2019-07-26

**Authors:** Paola E. Cuartas-Otálora, Juliana A. Gómez-Valderrama, Andrea E. Ramos, Gloria P. Barrera-Cubillos, Laura F. Villamizar-Rivero

**Affiliations:** 1 Corporación Colombiana de Investigación Agropecuaria-Agrosavia, Centro de Investigación Tibaitatá, kilómetro 14 vía Mosquera-Bogotá, 250047 Cundinamarca, Colombia; 2 AgResearch, Forage Science, Lincoln Research Centre, Private Bag 4749, Christchurch 8140, New Zealand

**Keywords:** biopesticide, enhancement, Granulovirus, Nucleopolyhedrovirus, pathogenicity, *Spodoptera frugiperda*, virulence

## Abstract

The ability of the isolate VG008 of S. frugiperda granulovirus (SpfrGV) to enhance the infectivity of the isolate SfCOL of S. frugiperda multiple nucleopolyhedrovirus (SpfrMNPV) was evaluated on *S. frugiperda* larvae. Bioassays were performed with mixtures by using different proportions 90%:10% (M1), 95%:5% (M2) and 97.5%:2.5% (M3) of SfCOL:VG008, respectively. All mixtures showed higher insecticidal activity that SfCOL. The mixture M3 showed the highest enhancement of SfCOL reducing 11.40 times the Mean Lethal Concentration and 96 h in the Mean Time to Death. The enhancer activity of proteins derived from VG008 (GVPs) were also evaluated in mixture with SfCOL. The GVPs increased 27% larval mortality caused by SfCOL and damaged the peritrophic membrane of *S. litura* larvae, suggesting that the key point in this enhancing activity is the initial step of the larva colonization, the midgut infection. M3 was formulated and evaluated under greenhouse conditions in maize plants using different doses. The highest efficacy was obtained with the highest dose of M3 (8 × 10^11^ OBs/ha), which was similar to that found when formulated SfCOL was applied using an approximately twofold higher dose. The viral mixture M3 was selected as the active ingredient for developing a new biopesticide for a more efficient management of the pest in the field.

## 1. Introduction

The fall armyworm, *Spodoptera frugiperda* (J.E. Smith, 1797) (Lepidoptera: Noctuidae) is a polyphagous migratory pest, endemic to the western hemisphere, considered the most important pest in maize (*Zea mays* L) in Colombia [1] and the Americas. The control of *S. frugiperda* in this crop is mainly achieved using chemical insecticides, which are not specific and present high toxicity. In consequence their continued use can lead negative effects such as development of resistant populations, reduction of beneficial insects and adverse environmental impact by the accumulation of toxic residues in food chain and water and soil pollution [2].

To reduce these effects and maintain pest levels below the economic threshold, some strategies as biological control has been developed through the use of entomopathogenic viruses, mainly from *Baculoviridae* family or baculovirus (BV), which are viruses with high insecticidal activity and with a narrow spectrum of hosts [2]. *Baculoviridae* family includes insect-specific viruses with large, circular, dsDNA genomes between 80 to 180 kpb. The family includes four genera: *Alphabaculovirus* (lepidopteran-specific nucleopolyhedrovirus (NPV)), *Betabaculovirus* (lepidoteran-specific granulovirus (GV)), *Gammabaculovirus* (hymenopteran-specific NPV) and *Deltabaculovirus* (dipteran-specific NPV) [3].

Worldwide, different isolates of Spodoptera frugiperda Nucleopolyhedrovirus (SpfrMNPV) have been used for biological control of fall armyworm, with efficacies higher than 80% [4,5,6], demonstrating its potential to control the pest. For developing efficient biopesticides to control *S. frugiperda*, some strategies of insecticidal activity enhancement have been evaluated. Within these approaches, biological enhancers have demonstrated the ability to improve the insecticidal activity of BVs increasing the host susceptibility and decreasing the time of action [7].

In this sense, in previous works two baculoviruses—a nucleopolyhedrovirus and a granulovirus—were isolated from *S. frugiperda* larvae collected in a pasture crop in Colombia, suggesting a possible natural co- infection [8]. The SpfrMNPV (SfCOL) and the SpfrGV (VG008) were characterized morphologically, biologically and molecularly [8,9,10,11]. The gene content showed that SpfrGV VG008 possesses encoding sequences for virulence factors such as 3 chitinases and 2 enhancins [11]; this observation offers the possibility of postulating the use of this granulovirus as an enhancing factor for bio-insecticides based on SpfrMNPV for the control of *S. frugiperda*.

Early studies on other lepidopteran pest showed that mixtures between NPVs and GVs significantly increased the infectivity and virulence of the NPV over its natural host [12]. This effect was attributed to the virulence factors as proteins such enhancins and chitinases [7,13]. This enhancer effect has been related with the affinity of these proteins for insect intestinal mucin and chitin, proteins associated with the peritrophic membrane (PM), the first barrier of defense in lepidopteran larvae. This interaction increases the susceptibility of the insect host to viral infection by altering the PM permeability [7].

The aim of the present work was to study the potential of SpfrGV (VG008) to enhance the insecticidal activity of SpfrMNPV (SfCOL) against *S. frugiperda* larvae, in order to select the most virulent active ingredient to develop a novel a biopesticide to control the fall armyworm.

## 2. Materials and Methods

### 2.1. Insect Rearing

Larvae of *S. frugiperda* were obtained from a laboratory colony established from eggs and larvae collected in a maize crop located at Tolima (Colombia). This colony was refreshed every six months (F6) by introducing insects collected in the field. Larvae were maintained at 25 °C and 60% relative humidity (RH), under a natural photoperiod of 12:12 h (light: dark) and were fed with a wheat germ-based semi-synthetic diet [14].

### 2.2. Virus Production, Purification and Quantification

Two Colombian isolates of *S. frugiperda* baculovirus were used, one SpfrMNPV denominated SfCOL and one of SpfrGV denominated VG008, obtained from infected larvae of *S. frugiperda* found in a pasture at department of Córdoba (Colombia) [8]. Production was made separately for each viral isolate by inoculating larvae of *S. frugiperda* (neonate larvae for NPV and second instar larvae for GV) using the droplet feeding method [15]. For this purpose, starved larvae of *S. frugiperda* were orally inoculated with a suspension containing 10^6^ occlusion bodies (OBs)/mL, then larvae were individually maintained at 25 °C and 60% RH, under a natural photoperiod of 12:12 h (light:dark) until death. OBs were extracted from dead diseased larvae by homogenizing cadavers in 0.1% (*w/v*) sodium dodecyl sulphate (SDS) solution and purified by filtration and differential centrifugations. GVs suspensions were quantified by absorbance measurements at 280 nm and extrapolated from a standard curve [9], and NPVs suspensions were quantified using a Neubauer hemocytometer (Hawksley Ltd., Lancing, UK) under a light microscopy at 40×.

### 2.3. Evaluation of Mixtures between Granulovirus and Nucleopolyhedrovirus

The Mean Lethal Concentration (LC_50_) of three mixtures prepared using different ratio SfCOL: VG008 were determined: 90% SfCOL: 10% VG008 (M1), 95% SfCOL: 5% VG008 (M2) and 97.5% SfCOL: 2.5% VG008 (M3). Each mixture was adjusted to five total concentrations of viral particles (CTot): 1 × 10^4^, 1 × 10^5^, 1 × 10^6^, 1 × 10^7^ and 1 × 10^8^ OBs/mL. Suspensions individually containing SfCOL and VG008 were prepared and adjusted to the same five concentrations. All final suspensions contained 4% (*w/v*) sucrose and 1% (*w/v*) blue food dye Tuska^®^ (blue No. 1 and blue No. 2). Pathogenicity and virulence were determined on *S. frugiperda* larvae by using the droplet feeding method [15]. The absolute control corresponded to larvae without treatment and the treated control corresponded to larvae fed only with sucrose and dye solution without containing OBs.

Three groups of 10 s instar larvae (3 replicates) starved for 12 h were inoculated with each viral suspension and individually transferred into 14 mL cups provided with natural diet (disinfected leaves of *Ricinus communis*) and reared at 25 °C and 60% RH, under a natural photoperiod of 12:12 h (light:dark).

Larval mortality was recorded daily for 15 days post-inoculation. Dead larvae were macerated in 1 mL of sterilized water and vortexed to mix, samples were then observed at 40× under light microscopy. Experimental design was completely random, with three replicates per treatments and the complete experiment was performed on three different occasions. Mortality data of the treatments were corrected with the mortality data from the control treatment. The LC_50_ and LC_90_ of each viral mixture were determined by using the logit regression of the Generalized Linear Interactive Modeling program (GLIM) [16]. For the comparison of LC_50_ values, parallelism of the calculated lines was checked by using the parallel-line assay option.

Relative potency (RP) for each mixture was determined according with SfCOL and calculated using the following formula: RP = LC_50_ SfCOL/LC_50_ mixture. Virulence expressed as the Mean Time to Death (MTD) was calculated by using the mortality data obtained during the pathogenicity bioassays with the concentration of 1 × 10^7^ OBs/mL. Time-mortality data were subjected to Weibull survival analysis using the GLIM program [16].

### 2.4. Assessment of Enhancer Activity of Proteins Derived from VG008

Proteins derived from VG008 (GVPs) were prepared by dissolving the OBs in Na_2_CO_3_ 0.5 M (pH 10) at 37 °C for 2 h. Then, the undissolved material was pelleted by centrifugation at 13,000 *g* for 20 min. The supernatant containing GVPs was collected and the proteins were electrophoresed in 14% SDS-PAGE with Coomassie blue staining.

To evaluate the enhancer activity of GVPs a bioassay was performed in *S. frugiperda* second instar larvae by using the droplet feeding method [15] as described above. Larvae were treated with the mixture containing SfCOL (1 × 10^6^ OBs/mL) and VG008 GVPs (200 μg/mL). Additionally, four control treatments were set up: i) Larvae fed only with the GVPs (200 μg/mL); ii) Larvae fed only with SfCOL (1 × 10^6^ OBs/mL); iii) Larvae fed with SfCOL diluted in Na_2_CO_3_ 0.5 M (1 × 10^6^ OBs/mL) prepared immediately before inoculation: iiii) Non-treated larvae (Control). Larval mortality was recorded 10 days post-inoculation. Mortality values recorded in the treatments were corrected with the mortality in the control using the Schneider-Orelli formula to express the result as percentage of efficacy (%) [17]. Results were analyzed with one-way ANOVA and Tukey test (95%) using Statistix 8.0.

To study the effect of the GVPs over the integrity of larval peritrophic membrane, *Spodoptera litura* four instar larvae were fed with GVPs (500 μg/mL) and processed 4 h post-feeding. For that the complete guts were dissected from the larvae and fixed in glutaraldehide in PBS (2.5%) by 24 h at 4 °C. After that, the PMs were extracted and washed with ultrapure water, followed by dehydration with ethanol 70% and 100%. The PMs were cut and the contents removed before sputtering with palladium. Observations and images were made on a scanning electron microscope JEOL JSM IT-300 (JEOL Ltd., Tokyo, Japan).

### 2.5. Characterization of Selected Mixture Formulated as Wettable Powder 

Mixture M3 (97.5% of SfCOL + 2.5% of VG008) selected for its higher insecticidal activity was formulated as a wettable powder (WP) and then characterized following the methodologies described by [6] and [18].

#### 2.5.1. Moisture Content

Three samples of 0.5 g were tested in a moisture analyzer OHAUS MB 45 at 100 °C for 10 min.

#### 2.5.2. pH

Three samples of 0.1 g were resuspended in sterile distilled water and pH determination was made with a calibrated potentiometer Consort C931.

#### 2.5.3. Contaminants Content

Three sample of 0.1 g were suspended in 0.5% (*v/v*) Tween 80 solution. Ten-fold dilutions were prepared, and 100 µL of each dilution were plated on Petri dishes containing Potato Dextrose Triton Agar (PDTA) medium and incubated five days at 26 °C to evaluate contaminant mold; on Yeast Malt Agar (YMA) plates incubated for two days at 26 °C to evaluate contaminant yeasts and on Nutrient Agar (NA) plates incubated for 24 h at 28 °C to determine the number of contaminant bacteria. Results were expressed as average number of colony forming units per gram (CFU/g) [19].

#### 2.5.4. Insecticidal Activity in Laboratory

*S. frugiperda* neonate larvae were inoculated with the viral mixture (formulated and unformulated) following the droplet feeding method [15]. Treatments were adjusted to a total concentration of 2 × 10^7^ OBs/mL containing 4% (*w/v*) sucrose and 1% (*w/v*) blue food dye Tuska^®^ (blue No. 1 and blue No. 2). Control treatments consisted in a treated control where larvae were inoculated with the sugar-colorant solution without viruses and an absolute control with non-treated larvae. Experimental design was completely random with three replicates of 10 larvae per treatment (30 larvae/treatment). Larvae were maintained at 25 °C and the number of surviving insects was recorded seven days after inoculation. Mortality values were determined and treatment mortality was corrected with the value in the control (larvae without treatment) by using the Schneider-Orelli equation [17].

#### 2.5.5. Photostability

Stability against ultraviolet (UV) radiation was determined following the methodology described in [20]. Mixture M3 and SfCOL formulated and unformulated were reconstituted in sterile water adjusting a final concentration of 2 × 10^7^ OBs/mL. Samples of 20 μL of each viral suspension were placed in four continuous microplate wells (each treatment in one row). Before irradiation, the first well of each row was covered with aluminum film in order to protect it against UV light, corresponding these samples to non-irradiated treatments. Then, the microplate was located inside a chamber at 10 cm distance from a Repti Glo 8.0 lamp which simulates solar UV radiation (33% of UVA and 8% of UVB) and irradiated for 6 h. Each 2 h after irradiation was initiated, the next well of each row was covered with aluminum film, corresponding to a different irradiation time. After the exposure to UV light, all samples were collected and evaluated for viral activity in a bioassay following the methodology previously described. 

### 2.6. Greenhouse Trial to Select Application Dose

Insecticidal activity of formulated mixture M3 was evaluated on maize plants under glass greenhouse conditions at the Tibaitatá Research Center, Mosquera, Cundinamarca (Colombia) (4°41’45”N, 74°12’12”W). Maize seeds (ICA 508) were planted individually in 16 ounces pots with soil. Thirty days after plants emergence when the plants had around 20 cm height and three true leaves, treatments were applied. Each plant was sprayed with 1 mL of the respective treatment by using a manual atomizer and 15 min after application when leaves were dry, three second instar larvae of *S. frugiperda* were placed on each plant.

Treatments were the formulated mixture M3 adjusted to five different doses (Table 1) and formulated SfCOL adjusted to its recommended dose [8]. Control treatment consisted in non-treated plants.

Experimental design was randomized complete blocks, with three replicates per treatment and experiment was repeated twice in different times. Each experimental unit consisted of ten plants, with a total of 30 plants per treatment. Four days after application, larvae were recovered, counted and individually placed in plastic cups with natural diet (*R. communis* leaves). Larvae were reared at 25 °C and the number of surviving insects was recorded during the next 15 days. Only individuals with viral infection symptoms were recorded to determine mortality values, which were corrected with the mortality in the control (larvae without treatment) by using the Schneider-Orelli equation [17].

## 3. Results

### 3.1. Mixtures between Granulovirus and Nucleopolyhedrovirus

**Figure 1:**
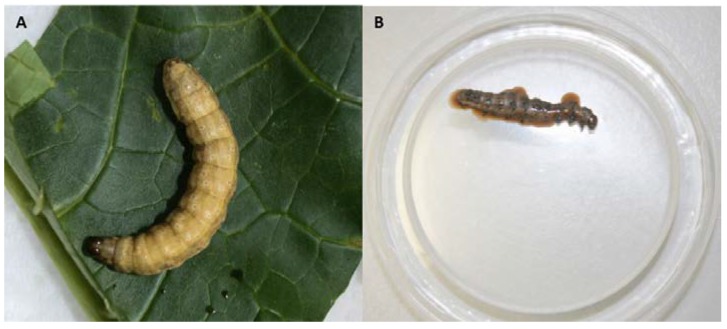

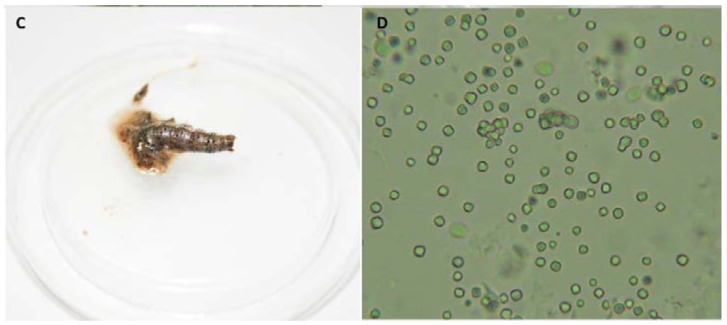
Larvae of *S. frugiperda* infected with (**A**) VG008 at 29 days post-infection, (**B**) SfCOL at 7 days post-infection, (**C**) mixture M3 after 5 days post-infection, and (**D**) nucleopolyhedrovirus occlusion bodies observed in macerates of *S. frugiperda* larva infected with mixture M3, examined under light microscopy (40×).

To determine an enhancer effect of VG008 on biological activity of SfCOL against second instar larvae of *S. frugiperda*, a dose-response bioassay with several mixtures was conducted. Larvae infected with VG008 slowly developed the disease and survived for several weeks, developing the typical symptoms of a GV infection, i.e., loss of appetite, decrease in mobility, yellowish-white coloration and a swollen larva body due to the accumulation of occlusion bodies in the infected tissues, with non-ruptured integument after dead (Figure 1A). Larvae infected with SfCOL rapidly developed symptoms of NPV infection with brownish color, waxy appearance and liquefaction of the cadaver (Figure 1B). Most larvae infected with the viral mixtures exhibited symptoms of NPV disease and high polyhedral production in the cadavers was confirmed by examination under light microscopy (40×) (Figure 1C,D).

Generated logit regressions showed a direct relationship between dose and mortality. The LC_50_ value for SfCOL was 2.05 × 10^5^ OBs/mL, which not significantly differed from the LC_50_ value for VG008 (Table 2). The LC_50_ values for mixtures ranged from 1.80 × 10^4^ OBs/mL for M3 to 5.87 × 10^4^ OBs/mL for M2 with no significant differences between them (Table 2). However, all mixtures presented significantly lower LC_50_ than SfCOL (*P* < 0.001), indicating higher pathogenicity (Table 2).

Parallelism of regression lines for the mixtures and SfCOL were determined to calculate the RP (Table 2). All combinations of VG008 and SfCOL were significantly more potent than the SfCOL used alone. Mixtures M1 and M2 were approximately fourfold more pathogenic than SfCOL. The most potent mixture was M3 (VG008 2.5% and SfCOL 97.5%), being approximately 12-fold more pathogenic than SfCOL.

Regarding the virulence, MTD of all mixtures ranged between 3 and 4 days, values significantly lower than the obtained for SfCOL and VG008 evaluated individually, with 7 and 29 days, respectively. Although differences were not detected between all mixtures, the lowest MTD was obtained with the mixture M3 (Table 2).

### 3.2. Enhancer Activity of Proteins Derived from VG008

**Figure 2:**
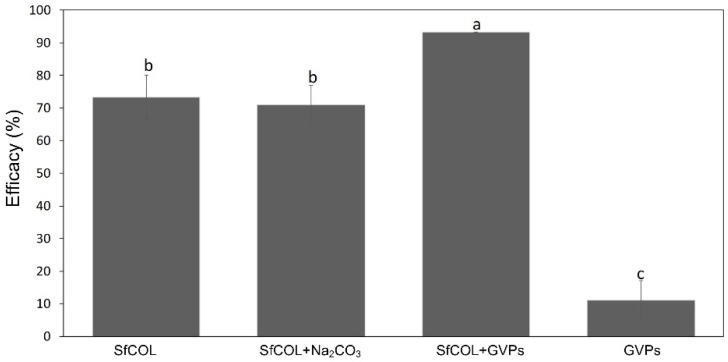
Effect of SfCOL and GVPs on *S. frugiperda* larvae mortality. Treatment mortalities were corrected with mortality in the control to estimate efficacy. Bars labelled with the same letters did not differ significantly according to Tukey test (95%). Error bars indicate the standard deviation (SD).

Feeding *S. frugiperda* L2 larvae with 1 × 10^6^ SpfrMNPV OBs/mL resulted in 73.3% efficacy (Figure 2). When the same viral suspension was mixed with GVPs adjusted to a final concentration of 200 µg/µL, the efficacy increased to 93.3% (Figure 2). The differences are statistically significant (*P* < 0.001). Non-significantly differences were found between efficacy obtained with SfCOL and SfCOl mixed with Na_2_CO_3_ (71.1%) and efficacy caused by the GVPs was significantly lower than that obtained with all the other treatments (11.1%) (Figure 2). Mortality was not observed with the non-treated larvae.

With the aim to demonstrate the presence of possible proteins enhancing the NPV, the proteins released after alkaline treatment of VG008 OBs were electrophoresed. The SDS-PAGE of GVPs showed different bands corresponding to estimated sizes of viral proteins previously reported in the complete genome of VG008 [11,21]

**Figure 3:**
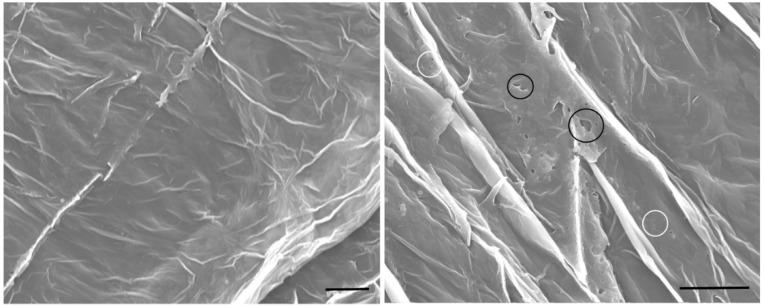
Scanning electron micrographs showing the effect of GVPs on *S. litura* peritrophic membrane (PM). (Left) PM of control larvae. (Right) PM of larvae treated with GVPs. White circles indicate small holes and black circles indicate larger holes. The black line corresponds to 10 µm.

The peritrophic membrane of *S. litura* larvae fed with GVPs showed damages in the structure (Figure 3). The surface presented several small holes (200–600 nm), which seem to be joining together at the most affected regions to form larger holes up to 2 µm.

### 3.3. Characterization of Selected Mixture Formulated as Wettable Powder

The characteristics of the prototype based on viral mixture M3 formulated as WP are presented in Table 3. Although there are no quality standards for baculovirus products that are internationally accepted [22], the results were compared with acceptance limits established in [6] for formulated SfCOL, to ensure that the formulation does not affect the characteristics of the viral mixture.

Physicochemical properties as moisture content and pH were within the reference limits, with values of 2.30 and 7.67, respectively. The bacterial and fungal contaminating load also met specifications, with values <10^7^ CFU/g. The efficacy evaluated in neonate larvae of *S. frugiperda* was 96.7% for M3 formulated as WP, equal value to that obtained with the unformulated viral mixture (Table 3).

Regarding photostability, the initial efficacies for non-irradiated viruses were 96.7% and 100% for mixture M3 and SfCOL respectively, with non-statistical differences between them or between formulated and unformulated viruses (*F* = 0.67, *df* = 3, 8, *P* = 0.5957) (Table 4). However, the efficacy of unformulated viruses progressively reduced as the irradiation time increased. The unformulated viral mixture M3 was rapidly and significantly inactivated after 2 h of irradiation, while the formulated mixture was stable and maintained its insecticidal activity through the 6 h of irradiation time with values above 96% of efficacy (Table 4). Similar result was obtained for SfCOL with 100% of efficacy when virus was formulated and irradiated for 6 h and a significant 90% drop in efficacy of unformulated virus after 2 h of UV-exposure (*F* = 18.4, *df* = 15, 32, *P* = 0.0000). The original activity remaining (OAR) of unformulated M3 and SfCOL after 2 h UV-irradiation was 6.9% and 10% respectively, without differences between treatments suggesting that both active ingredients (unformulated viruses) are equally susceptible to UV-radiation (Table 4). The OAR values of formulated viruses were significantly higher (*F* = 16.4, *df* = 11, 24, *P* = 0.0000) than those obtained with unformulated treatments at all evaluated times for SfCOL and the viral mixture M3 (Table 4).

### 3.4. Greenhouse Trial to Select Application Dose

Four days after infestation of the maize plants with larvae, the typical signs of damage caused by *S. frugiperda* were observed in all maize plants, such as translucent spots and small, irregular perforations. Larval mortality in the untreated plants (control treatment) was 9.4%, a significantly lower value (*F* = 41.6, *df* = 6, 35, *P* = 0.0000) compared with all the viral treatments, except that obtained with the lowest dose of the M3 mixture (5.0 × 10^10^ OBs/ha) (Table 5).

Mortalities and efficacies obtained with the viral treatments were statistically different between the evaluated doses (*F* = 26.3, *df* = 5, 30, *P* = 0.0000) (Table 5), showing a directly proportional relationship between the dose and the mortality. The highest efficacy was obtained with the highest dose of mixture M3 (8 × 10^11^ OBs/ha), which was similar to that found when formulated SfCOL (SfMNPV) was applied using an approximately twofold higher dose (Table 5).

## 4. Discussion

In this study, the increase of insecticidal activity of SpfrMNPV by adding low ratio proportions of SpfrGV over second instar larvae of *S. frugiperda* was studied. All larvae infected with viral mixtures (SfCOL + VG008) showed the typical symptoms of polyhedrosis disease, such as a characteristic shiny-oily appearance and extremely fragile tegument, which rupturing releases a fluid filled with virus particles. The combination of both viruses increased efficacy, and in the dead larva was possible to observe NPV OBs, but not GV OBs.

Mixture M3 (97.5% of SfCOL and 2.5% of VG008) presented the highest relative potency and virulence with the lowest MTD and significantly higher pathogenicity than observed with the wild isolates SfCOL and VG008 (Table 2), demonstrating that interaction between the viruses lead to an increase in efficacy. Additionally, when the GV proteins are added, this increase is maintained. Accordingly, it seems that the key point is the initial step of the larva colonization, the midgut infection. However, further histopathological studies are needed to elucidate the changes in the infection course caused by the mixture of VG008 and SfCOL.

Similar researches have reported different viral interactions between GVs and NPVs that improve the viral pathogenicity and virulence. For example mixtures between a GV and a NPV of *S. litura* showed a RP of 6.5 with respect to the pathogenicity of the NPV alone [23], the mixture of a GV and a NPV of *Spodoptera exigua* and a GV of *Xestia c-nigrum* and a NPV of *Mamestra brassicae* which presented a RP of 10 with respect to the LC_50_ of NPVs used alone [24,25,26]) and the mixture between a GV of *S. frugiperda* and a NPV of *Lymantria dispar*, being this mixture 13 times more pathogenic that the NPV individually used [27].

The mixture M3 presented the lowest MTD, with a value of 72 h (3 days), which means 96 h reduction in comparison with SfCOL and 624 h reduction in comparison with VG008. These results are higher than those achieved in other cases of mixtures of GVs and NPVs. For example, the mixture between the GV of *Trichoplusia ni* and the NPV of *Anticarsia gemmatalis*, the GV of *X. c-nigrum* and the NPV of *M. brassicae* and mixture between the GV of *Helicoverpa armigera* and the NPV of *L. dispar*, presented MTD reductions of 2, 48 and 55 h respectively [25,26,27,28,29]. Potential virulence factors of GV associated with an enhancer effect over NPV has been described, these factors include proteins contained in the occlusion bodies of different granulovirus isolates [23,26,27,30,31]. For example, alkaline soluble proteins of capsules (GVPs) of *X. c-nigrum* GV mixed with M. brassicae NPV, resulted in a significant increase of NPV infections (from 53% to 66%) under field conditions [30]. This enhancement has been attributed to proteins as enhancins and chitinases but also proteins with binding chitin capacity as *per os* infection factor 2 or GP37 [7,29,31,32,33]. These kinds of proteins can digest the proteins of the PM from infected insects by altering its structural integrity and increasing its permeability, allowing a faster entry of the viral particles into the cells improving the infection process [7,29,34,35].

It had been suggested that the coinfection of H. armigera GV and Helicoverpa zea SNPV over *H. zea* larva could inhibit the NPV replication by outcompeting [36]. In the SfCOL and SpfrGV coinfection, the dead larva presented typical symptoms of NPV disease and no evidence of GV replication was observed, suggesting that enhancement of NPV was a consequence of action of GV proteins contained in the OBs. The proteins derived from VG008 OBs increased 27% the larvae mortality caused by SfCOL NPV, demonstrating that they are responsible for this enhancing activity. Several studies using recombinants proteins have also demonstrated the enhancer activity of these proteins in different granulovirus coinfection [13,25,35,37].

The peritrophic membrane of most lepidopteran insects (type I) is a layer formed by chitin, mucopolysaccharides and proteins that protect the midgut epithelium against abrasive particles, digestive enzymes and pathogens [38]. This membrane has pores ranging between 21–29 nm [39] which allow a selective movement of small molecules [38]. PMs of *S. litura* larvae fed with GVPs of VG008 showed a damaged structure exhibiting the formation of different size pores that reached up to 2 µm in the most affected areas, suggesting the enzymatic action of viral enhancing factors or enhancins that are involved in degradation of mucin II in PMs [12]. The genome of VG008 contains enhancins vef2 (ORF127) and vef4 (ORF132) genes that encodes 867 and 857 amino acids (aa) proteins respectively with predicted weights close to 99.8 kDa [11], which were observed in SDS-PAGE and are homologous of those of H. armigera GV and X. c-nigrum GV enhancins, previously reported as enhancers of NPVs.

On the other hand, the enhancing action of chitin-binding proteins from granuloviruses that do not encode enhancin homologous proteins have been described, for example Epinotia aporema GV [31]. In this case, the GP37 protein seems to have a role in the disruption of PM by its chitin-binding properties [32]. The VG008 genome possesses chitinase-1 (ORF010), chitinase-2a (ORF071) and chitinase-2b (ORF072) and chitinase-2c (ORF134) smaller than baculoviral chitinases, but all of them with chitin-binding type-2 domains [11], which could act over the chitin of PMs. The results suggest that enhancer activity of proteins derived from occlusion bodies of S. frugiperda granulovirus VG008 on the infectivity of SpfrNPV SfCOL was due to the presence of enhancins and chitin-binding proteins that act over mucin II and chitin in the peritrophic membrane causing formation of holes that could ease the access of NPV virions to the epithelial cells of midgut and possibly other pathogens and digestive enzymes.

The prototype formulation using the mixture M3 presented adequate quality characteristics, for example the low moisture that will prevent the development of contaminants and favors the stability of the product during storage by reducing the microbial metabolism [40]. Likewise, the neutral pH guarantees the stability of viral particles because alkaline pH triggers the dissolution of OBs and the release of occluded virions [41]. Furthermore, [40] recommend that dry powder formulations should not exceed a maximum of non-pathogen contaminants of 5 × 10^8^ CFU/g. Baculovirus-based formulations with high level of contaminants usually present losses in virulence because the high metabolic activity of contaminating microorganisms that change the pH and cause the degradation of occlusion bodies [40,42] Therefore, the content of contaminants found in the WP-formulation based on mixture M3 can be considered low and could contribute to extend the shelf-life of the stored product.

Formulated and unformulated mixture M3 presented the same efficacy against *S. frugiperda* larvae, suggesting that the formulation process did not affect the biological activity of viral mixture. It should be noted that the insecticidal activity was above the minimum limit established for this type of products (>80%) [6], complying with the quality standards suggested to ensure high effectiveness in field applications.

The WP-formulation protected the viral particles against the adverse effects of UV-B radiation, due to the polymeric coating achieved by the microencapsulation process with Eudragit^®^ S100, as previously demonstrated in [6]. The formulated virus presented 79% efficacy and 14% inactivation after six hours of irradiation with a Repti Glo 8.0 lamp that simulates sunlight, while the unformulated virus drastically reduced its efficacy to 46%. The photo-stabilizer effect of this microencapsulated formulation was also evidenced in the present work with the viral mixture M3, demonstrating its efficiency to protect different viral active ingredients against the solar radiation. Solar radiation has been considered the most critical environmental factor for the survival of baculoviruses in the field, where the spectrum of ultraviolet light below 390 nm is responsible for the gradual inactivation of the virus [43,44].

Regarding the dose selected under greenhouse conditions, in a previous work [6] selected 1.5 × 10^12^ OBs/ha as the effective dose for field applications of the biopesticide based on SfCOL, which was used in the present work. Results suggested that changing the active ingredient of this developed WP-biopesticide from using SfCOL alone to using the mixture M3 could lead around 50% reduction in the minimum effective dose for field applications, which could contribute to overcome the limitations related to the costs of in vivo viral production and in this way increase the economic feasibility of this product. The reduction in the dose confirms the enhancing effect of GV on NPV infection, even under in plant conditions. Moreover, for other formulations based on SpfrNPV, as those developed with isolates from Nicaragua and the United States (3AP2), which have been evaluated in maize crops in Mexico, Honduras and Tanzania, the recommended application doses range between 1.2 × 10^12^ and 6 × 10^12^ OBs/ha [45,46], which are between 33% to 43% higher than the dose selected in this work for the biopesticide based in the mixture M3.

## 5. Conclusions

The results of this study demonstrated the enhancer activity of SpfrGV over the insecticidal activity of SpfrNPV. The low GV:NPV ratio in mixture M3 (97.5% of SpfrNPV and 2.5% of SpfrGV) exhibited the maximum enhancer potential by increasing the pathogenicity 9.92 times. This effect could be related with some proteins encoded by the Colombian isolate VG008 that are involved in the midgut infection. The mixture M3 was selected to develop a new and more efficient biopesticide to control the fall armyworm by applying lower doses in the field, helping to overcome the technical and economic limitations of baculovirus-based products and improving its economic feasibility.

## 6. Patents

*Virus based biopesticide*: Invention patent requested for United States, No. 15387565 [47].

## Figures and Tables

**Table 1:** Treatments evaluated in the greenhouse trial against *S. frugiperda*.

Treatments	Total Dose OBs/ha	Viral Mixture Composition	
		NPV OBs (97.5%)	GV OBs (2.5%)
M3 Dose 1	5.0 × 10^10^	4.9 × 10^10^	1.2 × 10^9^
M3 Dose 2	1.0 × 10^11^	9.8 × 10^11^	2.4 × 10^9^
M3 Dose 3	2.0 × 10^11^	2.0 × 10^11^	4.9 × 10^9^
M3 Dose 4	4.0 × 10^11^	3.9 × 10^11^	9.8 × 10^9^
M3 Dose 5	8.0 × 10^11^	7.8 × 10^11^	2.0 × 10^10^
SfCOL-F	1.5 × 10^12^	1.5 × 10^12^	-

**Table 2:** Estimated LC_50_ values, relative potencies (RP) and mean time to death (MTD) values of SfCOL and VG008 mixtures in *S. frugiperda* second instar larvae.

Treatment	LC_50_ (OBs/mL)	Fiducial Limits (95%)		RP	*P* Value	MTD (Days)	Fiducial Limits (95%)	
		Low	High				Low	High
SfCOL	2.05 × 10^5^	1.08 × 10^5^	3.81 × 10^5^	-	-	7	7	8
VG008	4.65 × 10^5^	1.91 × 10^5^	1.13 × 10^6^	0.44	0.110	29	25	33
M1	4.30 × 10^4^	2.51 × 10^4^	7.17 × 10^4^	4.76	<0.001	4	4	4
M2	5.87 × 10^4^	3.49 × 10^4^	9.77 × 10^4^	3.49	<0.001	4	4	4
M3	1.80 × 10^4^	1.01 × 10^4^	3.09 × 10^4^	11.40	<0.001	3	3	4

Logit regressions were fitted using GLIM program. A test for parallelism for all treatments was not rejected (*P* > 0.05). (*X2* = 4.66, *d.f.* = 3). Common slope (±SE) of 0.72 ± 0.08.

**Table 3:** Quality parameters of mixture M3 (97.5% of SfCOL + 2.5% of VG008) formulated as WP.

Parameter	Mean	SD	Acceptance Limits	Reference
Moisture content (%)	2.30	0.16	<5	[6]
pH	7.67	0.04	<8	
Bacteria content (CFU/g)	2.43 × 10^6^	0.07	<5 × 10^8^	
Mold content (CFU/g)	7.16 × 10^5^	0.12		
Yeast content (CFU/g)	<1 × 10^4^	0		
Efficacy (%)	96.7	5.8	≥80%	

**Table 4:** Susceptibility of unformulated and formulated mixture M3 to ultraviolet radiation.

Parameter	Irradiation Time (h)	Treatments			
		M3 F	M3 UF	SfCOL F	SfCOL UF
Efficacy (%)	0	96.7 a	96.7 a	100.0 a	100.0 a
	2	100.0 a	6.7 b	100.0 a	10.0 b
	4	96.7 a	3.3 b	100.0 a	6.7 b
	6	100.0 a	6.7 b	100.0 a	13.3 b
OAR (%)	2	100.0 a	6.9 b	100.0 a	10.0 a
	4	100.0 a	3.4 b	100.0 a	6.7 a
	6	100.0 a	6.9 b	100.0 a	13.3 a
Inactivation (%)	2	0 b	93.1 a	0 b	90.0 a
	4	0 b	96.6 a	0 b	93.3 a
	6	0 b	93.1 a	0 b	86.7 a

The statistical analysis was performed independently for each variable. Treatments with the same letter do not present significant differences according to multiple Tukey’s test (95%).

**Table 5:** Mortality of second-instar larvae of *S. frugiperda* under greenhouse conditions after the application of mixture M3 formulated as WP.

Treatment	Total Dose OBs/ha	Mortality (%)	Efficacy (%)
Control	-	9.4	-
M3 Dose 1	5.0 × 10^10^	28.6	21.2 c
M3 Dose 2	1.0 × 10^11^	37.8	31.3 c
M3 Dose 3	2.0 × 10^11^	62.6	58.7 b
M3 Dose 4	4.0 × 10^11^	67.4	64.0 b
M3 Dose 5	8.0 × 10^11^	91.5	90.6 a
SfCOL-F	1.5 × 10^12^	91.7	90.8 a

Treatments with the same letter do not present significant differences according to Tukey’s test (95%).
